# A Bidimensional System of Facial Movement Analysis Conception and Reliability in Adults

**DOI:** 10.1155/2015/812961

**Published:** 2015-06-16

**Authors:** Marjolaine Baude, Emilie Hutin, Jean-Michel Gracies

**Affiliations:** Laboratoire Analyse et Restauration du Mouvement, Service de Rééducation Neurolocomotrice, Hôpitaux Universitaires Henri Mondor, 51 Avenue du Maréchal De Lattre De Tassigny, 94010 Créteil, France

## Abstract

*Objective*. To design a bidimensional facial movement measuring tool and study its reliability.* Methods*. We utilized the free video-analysis software Kinovea that can track preselected points during movements and measure two-point distances off-line. Three raters positioned facial markers on 10 healthy individuals and video-taped them during maximal bilateral contractions of frontalis, corrugator, orbicularis oculi, zygomaticus, orbicularis oris, and buccinator, on two occasions. Each rater also analyzed the first video twice, one week apart. For each muscle, intrarater reliability was measured by percent agreements (PA) and intraclass correlation coefficients (ICC) between two assessments of the same video one week apart and between assessments of two videos collected one week apart. Interrater reliability was measured by PA, ICC, and coefficients of variation (CV) between assessments of the first video-recording by the three raters.* Results*. Intrarater and interrater reliabilities were good to excellent for frontalis (PA and ICC > 70%; CV < 15%), moderate for orbicularis oculi, zygomaticus, and orbicularis oris, and poor for corrugator and buccinators.* Discussion*. Without formal prior training, the proposed method was reliable for frontalis in healthy subjects. Improved marker selection, training sessions, and testing reliability in patients with facial paresis may enhance reliability for orbicularis oculi, zygomaticus, and orbicularis oris.

## 1. Introduction

Peripheral facial paresis following facial nerve injuries (traumatic, infectious, tumoral, autoimmune, and postneurosurgery) or conditions such as stroke, multiple sclerosis, myasthenia, and parkinsonian syndromes causes facial movement impairment that might be important to quantify for purposes of refined diagnosis or follow-up. In the management of peripheral facial paresis, a number of assessment methods have been proposed, initially by surgical teams and later also by rehabilitation physicians [1–8]. Among these, subjective clinical assessments comprise facial grading scales such as the House-Brackmann or Sunnybrook scales [9–12]. Objective quantitative facial assessments, using bidimensional and three-dimensional measurements, have often focused on one or very few facial muscles, omitting the rest of facial mobility [13–15]. Bidimensional techniques use photography or videography to measure distance between facial points at rest and during movement [15–24]. Three-dimensional assessments have used automation technologies and sophisticated algorithms, often to the cost of time-consumption, expensive equipment, and uneasy applicability to daily practice [25–28].

A quantitative facial movement assessment tool that would be easy-to-reproduce, fast, free, accurate, and reliable for a sufficient number of muscles remains an unmet need. Such a tool might help clinicians to quantify facial paresis at onset, during follow-up, and after interventions such as medical, surgical, and rehabilitative programs. In the present study we have used the free and open-source software Kinovea and selected specific facial markers to quantify movements of six key muscles. From standard subject videos, we measured normal resting facial distances and maximal excursions of the selected markers during movement. We explored the intrarater and interrater reliability of this method.

## 2. Methods

### 2.1. Subjects, Raters, and Procedures

**Figure 1:**
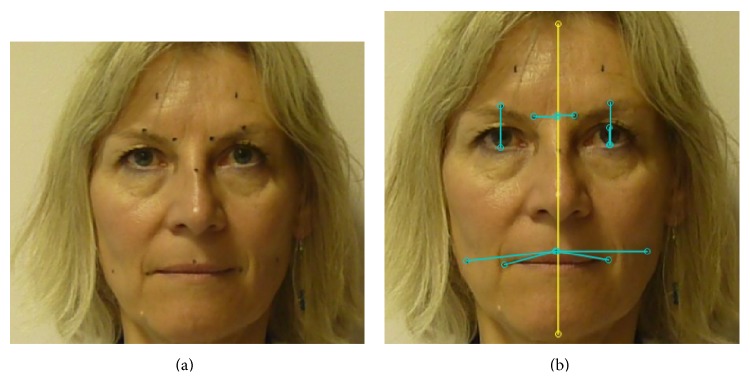
Principle of the Kinovea-derived method. Rest. (a) Each dot was drawn on the subject's face with face paint, corresponding to the preselected markers. The two dots on the forehead are 5 cm apart and are used for calibration. (b) Each line indicates distance measurements corresponding to the 6 selected muscles at rest. The lines from mid upper lip to the cheek point 4 cm out are used for both buccinator and orbicularis oris measurements.

The following procedures were administered in compliance with the Helsinki convention. Ten healthy subjects (6 women; age 39 ± 12) with no cervicofacial injuries or neurologic disorders participated in the study. Three raters (two physicians and one occupational therapist) who underwent short training about the method before used face paint to draw dots on the face of each subject on 10 preselected anatomic facial markers (Figure 1(a)):

- one at nasion (fixed marker);
- one at mid-upper lip;
- one at each mid eyebrow;
- one at each inner eyebrow tip;
- one at each mid-upper and each mid-lower lid;
- one at each oral commissure;
- one at a cheek point 4 centimeters from each oral commissure on the line from oral commissure to the mandibular angle.

To calibrate distances, two dots 5 centimeters away were also painted on the forehead.

Using these markers, we quantified 6 movements that each subject was to perform bilaterally using maximal contractions:

- raising eyebrows (frontalis muscle);
- frowning (corrugator muscle);
- eye closure (orbicularis oculi muscle);
- smile (zygomaticus muscle);
- puffing (orbicularis oris muscle);
- cheek incursion with attempted blowing (buccinator muscle).

### 2.2. Head Position and Movements

Facial movements were measured while subjects were comfortably seated on a fixed stool, with the upper buttocks, scapulas, and occiput leaning back against a wall. Subjects looked straight ahead towards a specified target fixed on the facing wall and were asked not to move during video acquisitions. The head was to be kept resting against the wall, at rest and during the 6 tested movements. Video-recording was performed at rest and during the 6 bilateral maximal facial contractions. Standardized, straightforward verbal commands were used for brow elevation (“raise your eyebrows”), frowning (“frown”), eye closure (“close your eyes”), smiling (“smile, showing your teeth”), puffing cheeks (“blow your cheeks keeping the air inside”), and cheek incursion (“bring your cheeks in”), using additional mimicking by the investigator as needed.

### 2.3. Kinovea Software

**Figure 2:**
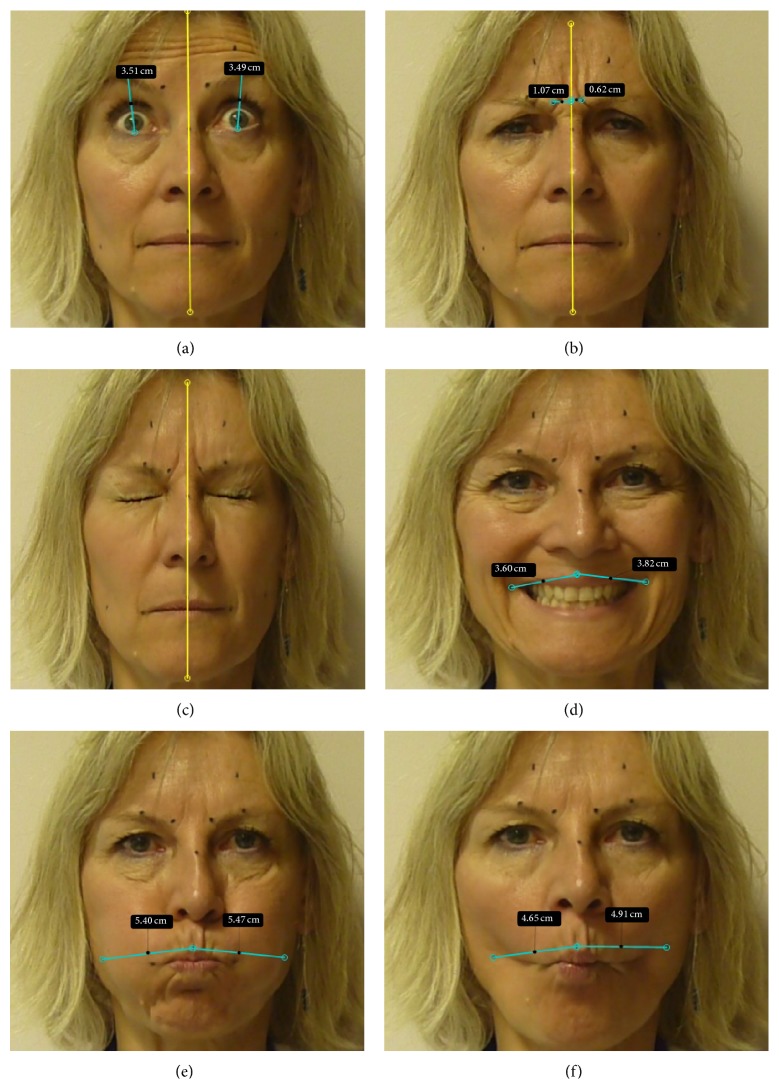
Principle of the Kinovea-derived method. Maximal contractions. Each blue line indicates distance measurements corresponding to the selected muscles during maximal contractions: (a) frontalis; (b) corrugator; (c) orbicularis oculi; (d) zygomaticus; (e) orbicularis oris; (f) buccinator. Note in (e) and (f) that in the subject selected the two cheek markers 4 cm out from the oral commissure fail to capture the maximum lateral cheek in/excursions.

Kinovea is a free and open-source (GPL2) French software created in 2009 as a tool for movement analysis (Kinovea, 0.8.15; Copyright © 2006–2011, Joan Charmant & Contrib, http://www.kinovea.org/) [29, 30]. Its straightforward functionalities are targeted to both movement science specialists and clinicians such as physical, occupational, or speech therapists. From plain video-recordings of movements, the software allows measuring distances and times, manually or using semiautomated tracking to follow points and check live values or trajectories. To our knowledge, Kinovea has not been used for facial analysis to date.  Figure 1(b) shows facial distances measured at rest using the software. Figures 2(a) to 2(f) show facial distances measured after the movements caused by maximal contractions of 6 selected facial muscles: frontalis (Figure 2(a)), corrugator (Figure 2(b)), orbicularis oculi (Figure 2(c)), zygomaticus (Figure 2(d)), orbiculari oris (Figure 2(e)), and buccinator (Figure 2(f)).

### 2.4. Assessment Procedure

All videos were analyzed using manual importing of the videos into the Kinovea software and calibrating each video to the 5 centimeter mark painted on the forehead of each subject (Figure 1(a)). A vertical midline was drawn through the nasion and mid-upper lip points to facilitate measures of corrugator movements (Figure 1(b)). The time to draw markers and perform each video acquisition was recorded, as well as the time to perform analysis using Kinovea. Measurements were taken on both sides of the face.

### 2.5. Statistical Analysis

Intrarater reliability was assessed for two different procedures, video analysis and marker positioning. First, we measured the intrarater reliability for video analysis (“interreview”) by calculating intraclass correlation coefficients and agreement frequencies between distances measured twice one week apart from the same video acquisition, for each muscle on each side. Then, we measured the intrarater reliability for marker positioning (“intermarking”), by calculating intraclass correlation coefficients and agreement frequencies between the distances measured in two video acquisitions performed one week apart for each patient, for each muscle on each side. For a given muscle, agreement was defined as a difference between two measurements equal to or lower than 20% of the mean distance measured across all subjects and raters over that movement (see Table 1). The level of agreement was defined as excellent above 85%, good between 70% and 85%, insufficient between 50% and 70%, and poor below 50%. To assess interrater variability we calculated intraclass correlation coefficients and agreement frequencies between distances measured by each rater from the first video acquisition, in addition to coefficients of variation (ratio of the standard deviation to the arithmetic mean) of the values between the three raters [31].

## 3. Results

The 10 healthy individuals who participated in the study were 6 women and 4 men, mean age 39 ± 12. All the videos acquired were deemed acceptable for analysis by the Kinovea software. In particular, there was no major head rotation noted from the plane of the camera.

### 3.1. Time Consumption

The entire acquisition, including marker painting, subject positioning, video-taping during rest, and the 6 maximal bilateral facial contractions, and marker removal took 4.0 ± 0.2 minutes (mean ± SD) to perform. Video-analysis took 20 ± 2 minutes for each video.

### 3.2. Raw Measurements

Table 1 shows the mean excursions of the selected markers in our subject group and the side-to-side symmetry ratios for each muscle on the first analysis of the first video (mean of 3 raters and 10 patients). The mean marker excursions covered by the different muscles ranged from 0.40 cm (left orbicularis oculi) to 1.36 cm (left frontalis); symmetry between right and left remained beyond 90% for upper face muscles and beyond 80% for lower face muscles.

### 3.3. Intrarater Reliability

**Figure 3:**
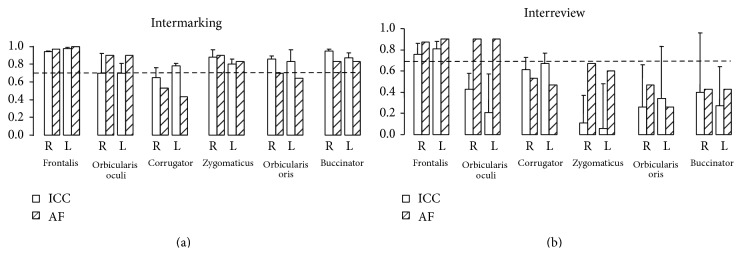
Intrarater reliability of the Kinovea-derived method. (a) Interreview corresponds to the reliability of two consecutive measurements, when the same rater reviews the same video twice one week apart. The dashed line at 0.7 represents the threshold for good to excellent reliability. ICC: intraclass correlation coefficients; AF: agreement frequency; R: right; L: left. (b) Intermarking corresponds to the reliability of two measurements made by the same rater using two different marker positioning sessions one week apart.

Figures 3(a) and 3(b) display the mean intrarater ICC (with standard deviation) and agreement frequencies (AF) per muscle on each side, between two video-analyses from the same marker positioning (“interreview”, Figure 3(a)) and between analyses from two different markings made one week apart (“intermarking”, Figure 3(b)). Regarding interreview reliability, both ICC and AF were good to excellent (>70%) for frontalis, orbicularis oculi, zygomatics, and buccinator; for corrugator and orbicularis oris, only ICCs were also good to excellent. There was a clear right-left symmetry in the intrarater reliability of measurements for each muscle (Figure 3(a)).

When facial marking was performed on two different days, only frontalis measurements retained excellent intrarater reliability, as well as orbicularis oculi but for agreement frequencies only. The other 4 muscles, corrugator, zygomaticus, orbicularis oris, and buccinator (particularly the latter two), fall below 70% reliability whichever the parameter considered. A sharp discrepancy was noted between poor ICCs and much higher AFs for orbicularis oculi and zygomaticus (Figure 3(b)).

### 3.4. Interrater Reliability

**Figure 4:**
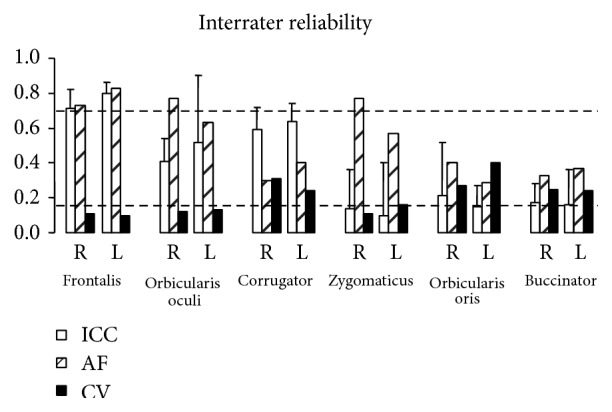
Interrater reliability. ICC: intraclass correlation coefficients; AF: agreement frequency; CV: coefficients of variation; R: right; L: left. The dashed line at 0.7 represents the threshold for good to excellent reliability and that at 0.15 represents the limit below which coefficients of variation are deemed acceptable.

Figure 4 displays the mean interrater ICC (with standard deviation), agreement frequencies, and coefficients of variation per muscle on each side. Interrater reliability was again good only for frontalis and questionable for orbicularis oculi and zygomaticus, these two muscles being characterized by small coefficients of variation (less than 16%) and agreement frequencies close to 70% on average, but by ICCs far below 70%.

## 4. Discussion

Despite substantial research on facial motion evaluation for the past decades, no single outcome instrument has become common practice among surgical or rehabilitation teams [14, 15, 17–28, 32–35]. This study shows that the first version of a method using the free and open-source Kinovea software applied without any prior formal training on plain video-recordings of facial movements was reliable for frontalis measurements. For zygomaticus and orbicularis oculi, reliability was suboptimal but might be expected to improve when examined in subjects with facial paresis because of higher intersubject variability in that population (see below). For the other tested muscles (corrugator, orbicularis oris, and buccinator), reliability was unacceptable with the current paradigm. Reliability improvement for these muscles might require refined marker selection and prior formal training before using the method. To best interpret the present findings, a number of methodological issues deserve discussion.

### 4.1. Intrarater Reliability: Interreview* versus* Intermarking

We broke down overall intrarater reliability into two components: the ability to agree with oneself when looking twice at a given video (“interreview” reliability) and the ability to agree with oneself when positioning markers twice on the same face (“intermarking” reliability). It must be acknowledged that the latter reliability measurement also involved two video-recordings and therefore also depended upon the first “interreview” reliability. Thus, the true “intermarking” reliability (or lack thereof) was really shown in the difference between the first and the second reliability, a difference that proved particularly obvious in some measurements for zygomaticus, orbicularis oculi, orbicularis oris, and buccinator (see Section 4.3).

### 4.2. Measures of Agreement, ICC* versus* Agreement Frequencies (AF)

Remarkable discrepancies were noted between AFs and ICCs on a number of occasions, in particular for intrarater intermarking and interrater reliability, regarding orbicularis oculi and zygomaticus on one hand (AF > ICC) and corrugator on the other hand (ICC > AF). One goal of this study was to answer two questions: “how often does a rater obtain the same results when looking at the same subject on two occasions?” (intrarater agreement rates, both interreview and intermarking) and “how often do two raters get the same result when observing the same subject?” (interrater agreement rate). The intraclass correlation coefficients answer a different question, which is a comparative one, as it is designed to compare the reliabilities of different tools used by the same group of raters on the same group of subjects [36]. The ICCs are thus devised to depend upon the homogeneity of the subjects used in a study [37–40]. Indeed, the ICC is the proportion of variability in all records, which is due to differences between subjects. This coefficient ranges from 0 to 1; the closer to 1, the more variability in the data comes from differences between subjects, the higher the agreement between raters or ratings. Mathematically, *ρ*_ICC_ = *σ*_*S*_^2^/(*σ*_*S*_^2^ + *σ*_*R*_^2^ + *σ*_*E*_^2^) where *σ*_*S*_^2^, *σ*_*R*_^2^, and *σ*_*E*_^2^ represent, respectively, the variance in the data that comes from the subjects, the rater, and random noise. For each muscle, these variances result from the fitting of the 2-way random effects ANOVA model: *x*_*i*,*j*_ = *μ* + *s*_*i*_ + *r*_*j*_ + *e*_*i*,*j*_ where *x*_*i*,*j*_ is the displacement measured on a given muscle of subject *i* by rater *j* (in this study, *j* = 1,2, 3 and *i* = 1,…, 10). *μ* is the average rating over all patients by all raters; *s*_*i*_ is the effect of subject *i* on rating, used as a random effect; *r*_*j*_ is the effect of rater *j* on rating, used as a random effect; and *e*_*i*,*j*_ is a random error. Formulas for its estimate, 95% confidence bounds, and the *F*-test for testing the null hypothesis of *ρ* = *ρ*_0_ are given in McGraw and Wong, 1996 (ICC [A,1], Case 2A model) [36]. The computation of that coefficient is thus meaningful as a comparative statistic between different measurement tools [36]. This was not the purpose of this study. To be clinically relevant we have thus opted to also report the agreement frequency, that is, the percent of matches, here defined as differences within 20% of the mean. Finally, when measuring interrater reliability of displacement measurements (Figure 4), we have additionally displayed the actual variability of ratings (coefficient of variation) between the 3 raters to complete the information.

A potential disadvantage of the agreement frequency method lies in the need for an arbitrary choice of a threshold difference below which “agreement” or “match” is defined. Here, our choice of a 20% difference for defining disagreement between two ratings corresponds to a range of differences from 0.8 to 2.7 mm depending on the muscles (20% of the mean distances covered, see Table 1). These differences are in fact small, as they fall within the parameters of facial asymmetry, which have been shown to be easily overlooked by human observers naive to the presence of a facial difference when asymmetry is less than 3 mm in the brow and oral commissure regions [41].

We thus elected to use the two statistics methods ICC and AF jointly and to compare their findings. In that respect, situations of frank discrepancies between the two reliability measurements may yield valuable information. For example, intermarking reliability for orbicularis oculi and zygomaticus was characterized by high agreement frequencies, while ICCs were low. This may have to do with high between-subject homogeneity of displacement values for these two muscles in a healthy population, which might lead to underestimate the reliability of the measure if using ICCs only. Such situation might be less likely to occur in a group of patients with peripheral facial paresis, in which differences from patient to patient would be expected to be higher than between healthy subjects moving all their facial muscles normally. Evaluations of the reliability of zygomaticus and orbicularis oculi measurements with the Kinovea-derived method in patients with facial paresis will be needed to confirm this hypothesis.

### 4.3. Muscle by Muscle Analysis, Marker Positioning Reliability

We initially selected 3 upper face muscles (frontalis, orbicularis oculi, and corrugator) and 3 lower face muscles (zygomaticus, orbicularis oris, and buccinator) to represent facial nerve function as extensively as possible. It is interesting to note that right-left symmetry was consistently about 10% higher for the upper face than for the lower face muscles, which is consistent with the bilateral descending innervation of upper face muscles only. Yet, varying degrees of reliability results for some of the selected muscles deserve analysis, particularly when comparing interreview and intermarking intrarater reliability.

For corrugator displacement, there was little loss of reliability between interreview and intermarking intrarater reliability, which may suggest that the issue may have to do with a difficulty in visually estimating the position of the markers on the inner angle of the eyebrow. Regarding zygomaticus and orbicularis oculi, the sole drops in ICCs from interreview to intermarking, which then dissociated from AFs, were discussed above. Orbicularis oris and buccinator might pose greater difficulties as Table 1 reveals standard deviations well beyond 20% of the mean in the estimations of their associated displacements, together with major discrepancies between interreview and intermarking intrarater reliability. This may reveal difficulty in finding reliable marker positions to reflect their associated movements (see examples of marker inadequation for orbicularis oris and buccinator in Figures 2(e) and 2(f)). In fact, the cheek displacements due to orbicularis oris and buccinator contractions are not only mediolateral but also anteroposterior and 3-dimensional technology might be more relevant to explore these muscles. Finally, the lack of previous training sessions might also have participated in high standard deviations for these 2 muscles in particular, as subjects had more difficulties in smiling or puffing than with the other requested movements. The reliability of frontalis measurements proved satisfactory probably because the marker positioning at mid eyebrow seems straightforward and its contraction-induced displacement occurs within a single frontal plan.

### 4.4. Limitations and Technical Issues, Head Movements, Choice of Marker Positions, Calibration, and Software Resolution

The first limitation is that this is not a study of the construct validity of the method. In other words, we have no information of systemic errors attached to the method [42]. Therefore additional studies will be required to deliver such information: how does this method compare to reference methods and actual measurements of the physical distance covered by cutaneous points during muscle contractions. Comparisons with 3D measurements in particular might be helpful in that respect.

Compensation for head movement with devices such as jigs or immobile reference points has been suggested, while many researchers consider on the other hand that restrictive fixation of the head or face may hamper natural facial movements [13, 32, 33, 43–47]. A number of measuring systems for facial motion analysis use markers that are attached to the facial skin instead of being painted as in the method described here. The use of physical markers in measuring systems is often time-consuming for both operator and patient, especially in 3-dimensional technologies. In addition, physical markers stuck on the face may alter or inhibit spontaneous facial motion. Some authors analyzed facial motion without markers [45] or positioned markers directly with the software [42]. Our choice of painted markers seems relevant because it is fast, cheap, and acceptable for individuals and operators.

Calibration scaling photographs to the iris diameter (11.8 mm in humans) have been reported [41, 42]. Here, a calibration using the distance nasion-tragus (not available except in 3/4 or profile incidences) as the more fixed points of the face could also be tested for comparison with our 5-centimeter frontal distance method. However, since at least one of the selected muscles proved to have very good reliability with all measures in the present study, calibration is probably not a critical issue here.

### 4.5. Comparison with Other Tracking Systems

In comparison with the available literature on bidimensional analysis, the presently described technique is free, open-source, fast to use, and presents with interesting advantages. In the system used by Hadlock and Urban [42] of a bidimensional Facial Assessment by Computer Evaluation (FACE) derived from Photoshop but using a MATLAB interface that allows faster analysis than the regular Photoshop technique the authors analyzed only 5 movements that were not specific of individual facial muscles and work on photographs only, as opposed to videos that we could freeze at the appropriate time of maximal muscle excursion, like with the present method.

To ascertain reliability for important muscles such as orbicularis oculi, zygomaticus, and perhaps corrugator, it seems important to reevaluate these muscles, together with frontalis, in patients with peripheral facial paresis. Such evaluation may be carried out without and with a formal prior training session for both patients and raters, and in parallel with clinical scales (Sunnybrook, Creteil) [48]. The case of buccinator and orbicularis oris is likely to need new marker selection to try to improve the Kinovea-derived method for these muscles.

### 4.6. Conclusion

A simple and easy-to-reproduce facial movement evaluation method has been designed using a free, open-source software to perform bidimensional analysis of movements related to 6 facial muscles. Without prior formal training, neither for subjects nor for investigators, intrarater and interrater reliability proved good to excellent in healthy subjects for the frontalis muscle only. For the other tested muscles, we may seek reliability improvement by refining the preselection of anatomic markers, by using formal training sessions for patients and raters and by testing the method in patients with facial paresis.

## Figures and Tables

**Table 1:** Distances covered and symmetry ratios (first analysis of the first video, mean of 3 raters, and 10 patients).

Muscles	Side	Mean distance (cm)	Standard deviation (cm)	20% distance (cm)	Minimum (cm)	Maximum (cm)	Symmetry ratio (%)
Frontalis	R L	1.32 1.36	0.26 0.28	0.26 0.27	0.59 0.66	1.77 1.87	97

Orbicularis oculi	R L	0.42 0.40	0.16 0.14	0.08 0.08	0.07 0.13	0.70 0.64	95

Corrugator	R L	1.07 1.08	0.15 0.15	0.21 0.22	0.80 0.80	1.32 1.36	99

Zygomaticus	R L	0.88 0.73	0.17 0.17	0.18 0.15	0.57 0.49	1.23 1.10	83

Orbicularis oris	R L	0.56 0.48	0.21 0.25	0.11 0.10	0.14 0.08	1.04 1.25	86

Buccinator	R L	0.70 0.87	0.29 0.29	0.14 0.17	0.25 0.32	1.26 1.53	80

Each number in Column 3 indicates the mean distance covered during displacement due to maximal contraction of the muscle indicated in Column 1. R: right; L: left. Note that the 20% difference selected to represent disagreement is lower than or equal to the standard deviation for all muscles except for corrugator.
